# Long-Term Care and Wide Effects on Children Caregivers -- New Evidence From Taiwanese Administrative Data

**DOI:** 10.1093/geroni/igab046.3623

**Published:** 2021-12-17

**Authors:** Kuan-Ming Chen, Chen-Wei Hsiang, Yu-Hsuan Chou, Shiau-Fang Chao, Kuan-Ju Tseng, Ming-Jen Lin, Ya-Mei Chen, Shih-Cyuan Wu

**Affiliations:** 1 National Bureau of Economic Research, CAMBRIDGE, Massachusetts, United States; 2 National Taiwan University, National Taiwan University, Taipei, Taiwan (Republic of China); 3 National Taiwan University, Taipei, Taipei, Taiwan (Republic of China); 4 National Taiwan University, Taipei City, Taiwan (Republic of China)

## Abstract

Long-term care (LTC) needs have profound impacts on the care-receivers and their children. Past research has focused on primary caregivers' short-term responses to LTC needs on limited aspects. This study brings new Taiwanese administrative data on health insurance, LTC program usages, and tax records. Rich information allows this study to explore long-term impacts on care-receivers' extended families. Event study combined with various regression analyses is the main framework of this study. Using the longitudinal record of more than 23 million individuals older than 65 over 18 years, the present study examines extended family members' various outcomes along the LTC needs trajectory. Among others, these outcomes include labor market participation and health expenditures. There are several findings in this study. Parents' LTC needs decrease all children's average full-time labor force participation by 2.5 percentage points even 10 years after the needs incurred. These needs do not directly increase children's health expenditure. A precise zero effect is found on children's health expenditure before, during, and after parents' LTC needs. Nevertheless, parents' health status or LTC risks, in general, may still pass on to children through other channels. The inter-generational health association is found to be approximately 0.25, indicating some degree of transmission. The results suggest that the impacts of LTC needs on family members are profound and widespread. Policy needs to address multiple aspects to cater to potential difficulties for care-receivers' family members.

